# Anemia in the Elderly After Renal Transplantation

**DOI:** 10.5812/numonthly.4507

**Published:** 2012-09-24

**Authors:** Somsri Wiwanitkit, Viroj Wiwanitkit

**Affiliations:** 1 Wiwanitkit House, Bangkhae, Bangkok, Thailand

**Keywords:** Transplantation, Anemia

Dear Editor,

The recent publication on anemia in the elderly after renal transplantation is very interesting (1). Rostami et al. concluded that “Anemia is very common in elderly patients undergoing kidney transplantation, particularly in patients with deteriorated allograft function (1).” The information from this report is very interesting. However, there are some concerns. First, the data is from a cross sectional descriptive study. The risk implication might be limited. There is also no data on the prevalence of anemia among these patients prior to renal transplantation. For sure, the pre-transplantation occulted anemia diseases, both genetic and non genetic ones, cannot be excluded. The good examples of underlying anemia are thalassemia and sickle cell anemia (2, 3). To conclude that anemia is adverse effect might not be suitable.
